# Sericin enhances ammonia detoxification by promotes urea cycle enzyme genes and activates hepatic autophagy in relation to CARD-9/MAPK pathway

**DOI:** 10.1016/j.heliyon.2023.e21563

**Published:** 2023-11-01

**Authors:** Sumate Ampawong, Napatara Tirawanchai, Tapanee Kanjanapruthipong, Kamonpan Fongsodsri, Khwanchanok Tuentam, Duangnate Isarangkul, Pornanong Aramwit

**Affiliations:** a Department of Tropical Pathology, Faculty of Tropical Medicine, Mahidol University, 420/6 Ratchawithi Road, Ratchathewi, Bangkok, 10400, Thailand; b Department of Biochemistry, Faculty of Medicine Siriraj Hospital, Mahidol University, 2 Wanglang Road, Bangkoknoi, Bangkok, 10700, Thailand; c Department of Microbiology, Faculty of Science, Mahidol University, 272, Rama VI Road, Ratchathewi, Bangkok, 10400, Thailand; d Center of Excellence in Bioactive Resources for Innovative Clinical Applications and Department of Pharmacy Practice, Faculty of Pharmaceutical Sciences, Chulalongkorn University, PhayaThai Road, Phatumwan, Bangkok, 10330, Thailand; e The Academy Science, The Royal Society of Thailand, Dusit, Bangkok, 10330, Thailand

**Keywords:** Ammonia detoxification, Hepatocyte, Pathology, Sericin, Urea

## Abstract

Urea cycle is an important metabolic process that initiates in liver mitochondria and converts ammonia to urea. The impairment of ammonia detoxification, both primary and secondary causes, lead to hyperammonemia, a life-threatening condition affecting to the brain. Current treatments are not enough effective. In addition, our recent proteomics study in hypercholesterolemic rat model demonstrated that sericin enhances hepatic nitrogenous waste removal through carbamoyl-phosphate synthase 1 (CPS-1), aldehyde dehydrogenase-2 (ALDH-2), and uricase proteins. However, the underlining mechanisms regard to this property is not clarified yet. Therefore, the present study aims to examine the effect of sericin on urea cycle enzyme genes (CPS-1 and ornithine transcarbamylase; OTC) and proteins (mitogen-activated protein kinase; MAPK, caspase recruitment domain-containing protein 9; CARD-9, Microtubule-associated protein light chain 3; LC-3), which relate to urea production and liver homeostasis in hepatic cell line (HepG2) and hypercholesterolemic rat treated with or without sericin. qRT-PCR, immunohistochemistry, and electron microscopy techniques were performed. *In vitro* study determined that high dose of sericin at 1 mg/ml increased liver detoxification enzyme (Cytochrome P450 1A2; CYP1A2 and ALDH-2) and urea cycle enzyme (CPS-1 and OTC) genes. Both in HepG2 cell and rat liver mitochondria, sericin significantly downregulated CARD-9 (apoptotic protein) expression while upregulated MAPK (hepatic homeostasis protein) and LC-3 (autophagic protein) expressions. Hence, it might be concluded that sericin promotes ammonia detoxification by both increases urea cycle enzyme genes and enhances hepatic autophagy in associated with CARD-9/MAPK pathway (as shown by their own negative relationship). This study presents another beneficial property of sericin to develop an upcoming candidate for ammonia toxicity alleviation and liver function improvement.

## Abbreviations

Alcoholic fatty liver disease: AFLD
Aldehyde dehydrogenase: ALDH-2
Carbamoyl-phosphate synthase 1: CPS-1
Caspase recruitment domain-containing protein 9: CARD-9
Central nervous system: CNS
Cytochrome P450 1A2: CYP1A2
Glyceraldehyde 3-phosphate dehydrogenase: GAPDH
Microtubule-associated protein light chain 3: LC-3
Mitogen-activated protein kinase: MAPK
Non-alcoholic fatty liver disease: NAFLD
Ornithine transcarbamylase: OTC
Sucrose phosphate buffer: SPB
Transmission electron microscope: TEM

## 1 Introduction

Urea cycle is an indispensable process to eliminate ammonia, a metabolic waste from amino acid catabolism in mammal, which takes place in the liver (both in hepatic mitochondria and cytoplasm) by convert ammonia to urea and excrete into the urine. Defect of ammonia detoxification or urea cycle dysregulation, either caused by inherited urea cycle disorder or liver diseases, leads to reversible or irreversible hyperammonemia, which mostly affects to central nervous system (CNS) causing to hepatic encephalopathy as shown by brain edema, coma, and convulsion [1,2]. Neuroprotective agent is needed for alleviation ammonia toxicity underwent their own related mechanisms for instance amino acid or neurotransmitter pathways, signaling transduction pathways, nitric oxide synthase pathway, and water channel pathway [1,2]. However, candidate therapeutic agents are still need to be developed.

Hepatic autophagy is a catabolic process for removing dysfunction cytosolic molecules. Up to date, impaired hepatic autophagy causes several acute or chronic liver diseases such as non or alcoholic fatty liver disease (NAFLD or AFLD) and hepatocellular carcinoma [[3], [4], [5]]. Interestingly, it is reported that hepatic autophagy accelerates a genesis of urea leading to hyperammonemia protection [6].

Mitogen-activated protein kinase (MAPK) signaling plays an important role in liver cell homeostasis such as redox balance and cell programming (survival, differentiation, proliferation, and senescence) [7]. Caspase recruitment domain-containing protein 9 (CARD-9), an apoptotic enhancer, also acts as a main role in metabolic processes both pathological and physiological aspects. It has been reported that CARD-9 polymorphism is related to the progression of liver diseases [[8], [9], [10]]. Furthermore, Caspase (e.g. -3, -9, or -10)/MAPK pathway also presents an advantage for explaining pathogenesis in several diseases focusing on specific tissue or cell alterations, recently in lung, kidney, gastro-intestinal tract, urinary bladder, and liver [[11], [12], [13], [14], [15]].

It is generally well known that sericin has its own biomedical prosperity such as blood cholesterol lowering effect [[16], [17], [18]], anti-melanogencity [19], anti-inflammation [20], and healing enhancement [21,22]. Interestingly, our previous report based on proteomics study in liver mitochondria extracted from hypercholesterolemic rat demonstrated that not only a regulatory effect of sericin on liver -apoptosis, -autophagy, -energetic balance, and -antioxidation, it also improves hepatic nitrogenous waste detoxification via carbamoyl-phosphate synthase 1 (CPS-1), aldehyde dehydrogenase (ALDH-2), and uricase [18]. However, the detail mechanisms of sericin in associated with ammonia removal or hepatoprotective property would not be clarified yet.

Along this line of thought, we hypothesized that sericin might be a new candidate for alleviation hyperammonemia with some underling mechanism regarding to its effects. Therefore, i*n vitro* study using liver cell line (HepG2) was conducted to explore, whether sericin promotes ammonia detoxification in relation to specific genes in liver detoxification and urea cycle (Cytochrome P450 1A2; CYP1A2, ALDH-2, CPS-1, and Ornithine transcarbamylase; OTC). Moreover, proteins (CARD-9, MAPK, and Microtubule-associated protein light chain 3; LC-3) that responsible for liver maintenance and urea synthesis were examined their expressions both in HepG2 cell and rat liver mitochondria to verify specific mechanism of sericin on ammonia removal capacity. This study could be able to point out another beneficial property of sericin for development an additional therapeutic product regarding to ammonia detoxification.

## 2 Materials and methods

### 2.1 Sericin extraction

Sericin was extract from the cocoon shells of *Bombyx mori* silkworm (Chul Thai Silk Co., Ltd., Petchaboon Province, Thailand) by autoclaving method. Briefly, the cocoon shells were autoclaved in purified water at 120 °C for 60 min and then filtrated. The extraction was aliquoted and kept in desiccator at room temperature. Amino acid content in the extraction was examined by Central Laboratory (Thailand) Co., Ltd using in-house method for Amino Acid Analyzer Technique and HPLC Technique based on Official Journal of the European Communities and Journal of Food Chemistry, respectively.

### 2.2 *In vitro* studies

#### 2.2.1 Cell culture

HepG2 cell line (ATCC, HB8065, VA, USA), were cultured in DMEM (Dulbecco's Modified Eagle Medium) supplemented with 10 % fetal bovine serum, 100 units/mL penicillin, and 100 μg/mL streptomycin at 37 °C in a humidified atmosphere of 5 % CO_2_ [[23], [24], [25]].

#### 2.2.2 Cell viability assay

To measure the effect of sericin on cell viability, HepG2 cells were seeded in 96 well plates for 24 h. The cells were treated with or without various concentrations of sericin (0, 0.125, 0.25, 0.5, and 1 mg/ml) and simvastatin (Merck, NJ, USA) (0, 0.01, 0.02, 0.04, and 0.08 mg/ml). After the exposure period, the medium was removed and MTT solution (5 mg/ml/well) was added for 4 h. Formazan was determined by dissolving in 100 μl of DMSO (dimethyl sulfoxide)/well and measured spectrophotometrically at 570 nm. The percentage of viable cells was calculated [[23], [24], [25]]. Appropriate concentration of sericin and simvastatin were selected for further experimental protocols.

#### 2.2.3 Experimental protocol

To determine whether sericin alters gene expression involving in urea cycle and as well as lipid and alcohol detoxifications, HepG2 cells were grown at a density of 10^6^ cells/ml and treated with 0.125 and 1 mg/ml of sericin and 2.5 μg/ml of simvastatin (regarding to the results from cell viability assay) for 24 h compared to non-treatment group. These concentrations were achieved from MTT assay [[23], [24], [25]]. The half maximal inhibitory concentration of sericin and simvastatin that calculated by the curve fit equations were 4.56 mg/ml and 0.02 mg/ml, respectively. End of the experiment, cells were trypsinized with 0.25 % Trypsin-EDTA, washed in phosphate-buffered saline, and centrifuged at 3500 rpm at 4 °C for 5 min. The HepG2 pellets were divided into 2 parts, one was kept in -80 °C for molecular technique and another was fixed in 2.5 % glutaraldehyde in sucrose phosphate buffer (SPB) for electron microscopic study.

#### 2.2.4 RNA isolation and quantitative reverse transcription-polymerase chain reaction (qRT-PCR)

After the treatment, total RNA samples extracted from treated cells were primarily transcribed into cDNA. Subsequently, expression of CPS-1, OTC, CYP1A2, ALDH-2 were quantified by qRT-PCR. Total RNA was isolated from HepG2 cells using TRIzol reagent (Invitrogen, Carlsbad, CA, USA) according to the manufacturer's instructions. RNA was reverse transcribed using RevertAid First Strand cDNA Synthesis Kit (Thermo Fischer Scientific, Waltham, MA, USA) according to the manufacturer's instructions. RT-qPCR was performed using the Luna® Universal qPCR Master Mix (Biolabs, MA, USA) according to the manufacturer's instructions. All specific primers used to amplify, CPS-1, OTC, CYP1A2, ALDH-2, and Glyceraldehyde 3-phosphate dehydrogenase (GAPDH) genes were designed by Oligo 7 primer analysis software (Table 1). Thermal cycle conditions were shown in Table 2. The Ct value was normalized using a house-keeping gene GAPDH. Relative gene expression was calculated using 2^−ΔΔCt^ method [25,26].

**Table 1:** Oligonucleotide sequences of the gene-specific primers used in qRT-PCR.

Genes	Sequence (5′-3′)
CPS1	F: TCAAGGCACAGACAGCACAC R: TTCATCCAGAGCAGTAGTATCAGG
OTC	F: GGACATTTTTACACTGCTTGCCC R: TCCACTTTCTGTTTTCTGCCTCTG
CYP1A2	F: AGCACAACAAGGGACACAACG R: ATGGCCAGGAAGAGGAAGAT
ALDH2	F: TTCGCCCTGTTCTTCAACCA R: CCTGCTCGGTCTTGCTATCAAA
GAPDH	F: CAGCCTCAAGATCATCAGCA R: CATGAGTCCTTCCACGATAC

**Table 2:** Thermal cycle conditions.

Gene	Cycle step	Temperature	Time	Cycle
CPS1	Initial Denaturation	95 °C	60 s	1
	Denaturation	95 °C	15 s	39
	Annealing	60 °C	60 s	
	Extension	72 °C	20 s	
OTC	Initial Denaturation	95 °C	60 s	1
	Denaturation	95 °C	15 s	39
	Annealing	58 °C	60 s	
	Extension	72 °C	20 s	
CYP1A2	Initial Denaturation	95 °C	60 s	1
	Denaturation	95 °C	15 s	39
	Annealing	60 °C	60 s	
	Extension	72 °C	20 s	
ALDH2	Initial Denaturation	95 °C	60 s	1
	Denaturation	95 °C	15 s	39
	Annealing	55 °C	60 s	
	Extension	72 °C	20 s	

### 2.3 *In vivo* study

#### 2.3.1 Ethical statement

Animal protocol was asked for a permission and approved by the Faculty of Medicine, Chulalongkorn University Animal Care and Use Committee, Bangkok, Thailand (Approval No. 16/2558). Sprague-Dawley rats, eight-week-old weighing 180–200 g, were acquired from the National Laboratory Animal Center, Mahidol University, Thailand. They were housed under standard conventional system with a condition of 12-h dark/light cycle and 25 ± 2 °C room temperature and provided by standard diet No. 082 (Perfect Companion Ltd., BKK, Thailand) and reverse osmosis water as *ad libitum*.

#### 2.3.2 Animal experimentation

Hypercholesterolemic condition was induced in the rats by feeding 6 % cholesterol-coated diet for 6 weeks as mentioned in Ampawong et., al, 2017 [16]. Then, they were divided into three groups (six rats of each) and orally gavaged with 2 ml/day of sterile water, 1000 mg/kg/day of sericin extract, and 7.4 mg/kg/day of simvastatin for 28 days to serving as non-, sericin-, and standard-treatment groups, respectively. After the rats were treated with any substance until endpoint, they were humanely euthanized with isoflurane® inhalation. Blood was collected from heart puncture. Total blood cholesterol was assessed by the Quality Control Division, National Laboratory Animal Center, Mahidol University, Thailand. Clinicopathological changes were performed.

#### 2.3.3 Tissue collection and mitochondrial extraction

The liver was removed and immediately kept in cooled mitochondrial extraction buffer (0.32 M sucrose, 1 mM EDTA and 10 mM Tris-HCl, pH 7.4). The liver mitochondria were extracted as our previous protocol [16,18,27,28]. Briefly, the liver was pooled, homogenized, and centrifuged at 1000 g 4 °C for 5 min to separate mitochondrial supernatant and liver tissue debris. Mitochondria were separated from the supernatant portion using centrifugation at 15,000 g and 4 °C for 2 min and four consecutively washed by extract buffer. The last pellets were fixed in 2.5 % glutaraldehyde in 0.1 M SPB for 1 h, washed in 0.1 M SPB for 10 min each and then kept in SPB at 4 °C.

### 2.4 Immunogold labelling

To identify the expression of associated proteins affecting to urea detoxification processes in both HepG2 cell and liver mitochondria in any group (non-treatment, simvastatin, and sericin), ultrastructural study was conducted as described by our previous studies [[16], [17], [18], [19],[27], [28], [29], [30], [31], [32]]. HepG2 cell and liver mitochondria pellets were primarily fixed in 2.5 % glutaraldehyde in 0.1 M SPB and secondarily fixed in 1 % osmium tetroxide in SPB, respectively. Samples were dehydrated in a series of ethanol, infiltrated in grading LR white resin (EMS, PA, USA), embedded in LR white, polymerized in 65 °C oven, and sectioned into 100 nm thick. Rabbit anti-caspase-9, -MAPK, and -LC-3 (MyBioSource, CA, USA) were applied as primary antibodies in immunogold labelling assay. Cells were blocked with 50 mM glycine and 5 % bovine serum albumin (BSA) (EMS, PA, USA) and then incubated with primary antibodies for 1 h at room temperature. Immunoglobulin (Ig) G conjugated with 10-nm gold particles (EMS, PA, USA) was applied to the sections for 1 h. Silver enhancement was performed using the Aurion R-Gent SE-EM kit (EMS, PA, USA). Finally, sections were stained with lead citrate and uranyl acetate and examined under transmission electron microscope (TEM) to assess the amount of gold labelling in the cellular area or mitochondria. Gold-labelled particles on HepG2 cell were counted for a whole cell containing 20 hepatocytic cells in each group/marker, while the labelling on liver mitochondria was considered by percentage of mitochondrial positive stain/field at 7000 × magnification.

### 2.5 Immunohistochemistry

To confirm the expression of LC-3 in the liver sections from rat-treated with sericin and simvastatin compared with non-treated rat, immunohistochemistry was performed as described by our previous studies [[16], [17], [18], [19],[27], [28], [29], [30], [31], [32]]. After deparaffinization with xylene and hydration with ethanol, the liver sections were retrieved antigenicity in citrate buffer (pH 6) using microwave, blocked the endogenous peroxidase activity and non-specific binding with 1 % v/v of hydrogen peroxide in methanol and 2 % v/v of bovine serum albumin (BSA; [EMS, PA, USA]) respectively. The sections were incubated with rabbit anti-LC-3, polymer HRP anti-mouse/rabbit labeling (Agilent Dako, CA, USA), diaminobenzidine visualization (Agilent Dako, CA, USA), and then counter-stained with hematoxylin. Finally, immunolocalization was examined under a light microscope.

The level of expression of each protein was measured in terms of the H-score (percentage area of expression × intensity score). ImageJ software was used to quantify the immuno-distribution area in terms of percentage. Color images (10 images/group) were captured at 400× magnification. The immunolabeled area was measured by a threshold mode to obtain the percentage of positive pixels after the conversion of images to grayscale. In addition, intensity was scored from 0 to 3 and was classified into four grading scales: 0–negative staining, 1–low-intensity staining, 2–moderate-intensity staining, and 3–high-intensity staining.

### 2.6 Statistical analysis

The comparison among groups was conducted by either non-parametric of independent *t*-test or analysis of variance depended on their data distribution using GraphPad® PRISM, version 6.05. The significant level was considered at *P*-value <0.05.

## 3 Results

### 3.1 Amino acid content

To verify the consistency of sericin extraction by lot to lot, amino acid profiles were examined as shown in Table 3. The ratio of Serine: Aspartic acid: Glycine was approximately to 3 : 2: 1, which is our in-house standardized criterion.

**Table 3:** Amino acid content of sericin extraction.

Amino acid profiles	mg/100 g extract	%
Aspartic acid	756.52	17.04446
Threonine	382.99	8.628797
Serine	1213.08	27.33079
Glutamic acid	307.73	6.933183
Glycine	439.81	9.908956
Alanine	195.8	4.41139
Valine	200.87	4.525618
Leucine	145.8	3.284886
Tyrosine	295.8	6.664399
Lysine	204.31	4.603121
Arginine	295.8	6.664399
Cystine	Not detected	0
Methionine	Not detected	0
Isoleucine	Not detected	0
Phenylalanine	Not detected	0
Histidine	Not detected	0
Hydroxylysine	Not detected	0
Hydroxyproline	Not detected	0
Proline	Not detected	0
Tryptophan	Not detected	0

### 3.2 High dose of sericin increased cytosol detoxification enzyme genes for lipid and alcohol metabolisms

**Fig. 1:**
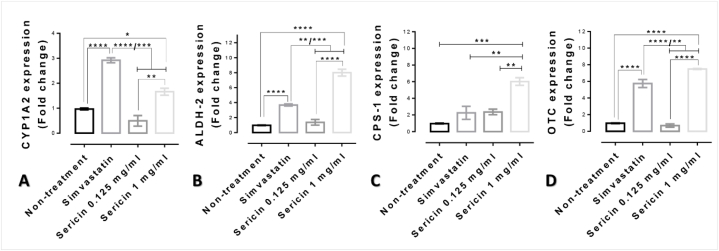
The level of cytosol detoxification enzyme and urea cycle enzyme genes in HepG2 cell among treatments: Bar graphs indicated the mRNA fold change expression of CYP1A2 (**A**), ALDH-2 (**B**), CPS-1 (**C**), and OTC (**D**) genes in HepG2-treated with or without simvastatin and two doses of sericin. *; *P* ≤ 0.05, **; *P* ≤ 0.01, ***; *P* ≤ 0.001, ****; *P* ≤ 0.0001.

The capacity of sericin on lipid and alcohol detoxifications was assessed by the expressions of CYP1A2 and ALDH-2 genes, respectively. Comparing with non-treated cell, fold change of expressions in any treatment was determined as shown in Fig. 1A-B. CYP1A2 gene expression was remarkably increased in 1 mg/ml sericin treated cell by 1.66-fold, whereas at 0.125 mg/ml of sericin treatment group was not detectable (Fig. 1A). In addition, simvastatin increased the CYP1A2 gene expression in HepG2 cell by 2.92-fold and had also significantly higher than presented in both concentrations of sericin-treated cells (Fig. 1A). In addition, 8-folds upregulation of ALDH-2 expression was observed in 1 mg/ml sericin-treated cell, which also significantly higher than exhibited in simvastatin-treated cell and other treatments (Fig. 1B).

### 3.3 High dose of sericin increased urea cycle enzyme genes in mitochondria

The effect of sericin on CPS-1 and OTC genes expression, the pivotal urea cycle genes at transcriptional level, was assessed (Fig. 1C-D). CPS-1 gene expression was significantly highest level in 1 mg/ml sericin-treated cell comparing with another-treated cell (up to 6.02-fold) (Fig. 1C). In contrast, the CPS-1 mRNA level in 0.125 mg/ml sericin-treated cell and simvastatin-treated cell were obviously low (Fig. 1C). Moreover, OTC gene expression was also evaluated. Although OTC gene expression in simvastatin-treated cell were significantly high when compared with non-treated cell and low dose of sericin-treated cell, it was significantly lower than presented in high dose of sericin-treated cell (Fig. 1D).

### 3.4 Sericin regulated CARD-9/MAPK pathway

**Fig. 2:**
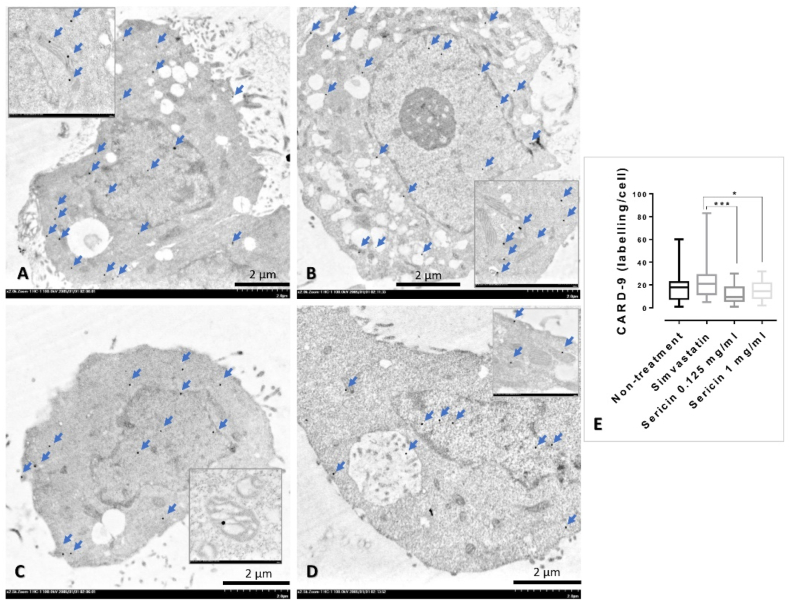
CARD-9 immunolabelling in the HepG2 cell among treatments: Electron micrographs shown immunogold labelling of CARD-9 expression (arrow) on HepG2 whole cell without (**A**) or with simvastatin (**B**) and sericin (**C-D**) treatments. CARD-9 immunolocalization was observed throughout of the HepG2 cell both in the cytoplasmic and nucleolar areas. Inset images represented a higher magnification with the expression on mitochondria and cytosol. Bar graph compared the expression among treatment groups (**E**). *; *P* ≤ 0.05, **; *P* ≤ 0.01, ***; *P* ≤ 0.001, ****; *P* ≤ 0.0001.

**Fig. 3:**
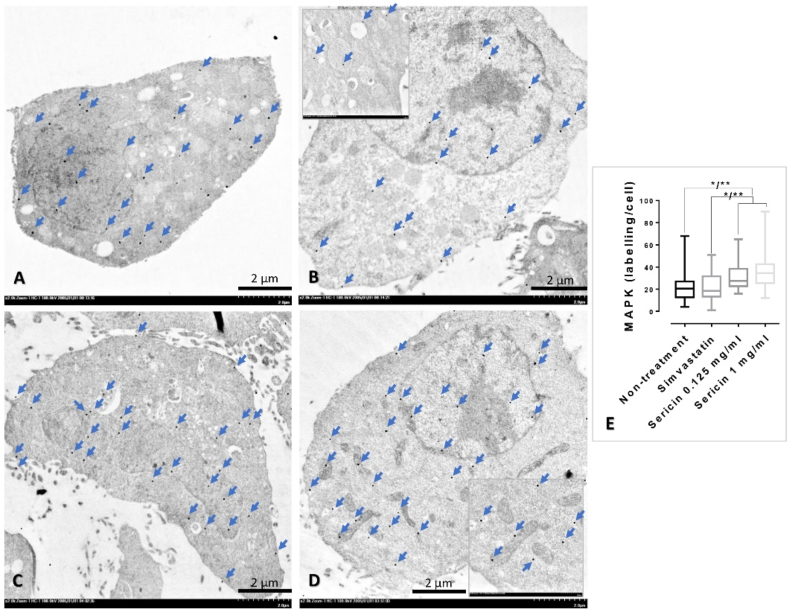
MAPK immunolabelling in the HepG2 cell among treatments: Electron micrographs shown immunogold labelling of MAPK expression (arrow) on HepG2 whole cell without (**A**) or with simvastatin (**B**) and sericin (**C-D**) treatments. MAPK immunolocalization was observed throughout of the HepG2 cell both in the cytoplasmic and nucleolar areas. Inset images represented a higher magnification with the expression on mitochondria and cytosol. Bar graph compared the expression among treatment groups (**E**). *; *P* ≤ 0.05, **; *P* ≤ 0.01, ***; *P* ≤ 0.001, ****; *P* ≤ 0.0001.

**Fig. 4:**
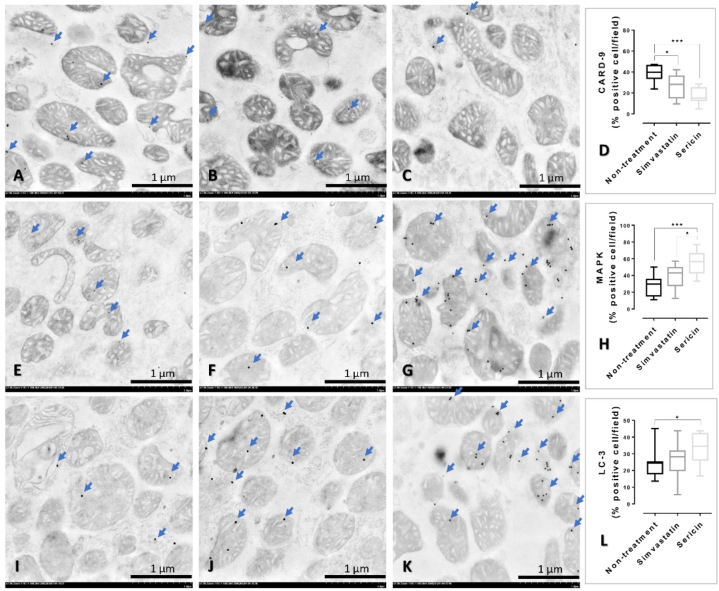
CARD-9, MAPK, and LC-3 immunolabelling in the liver mitochondria from the rat among treatments: Electron micrographs shown immunogold labelling of CARD-9, MAPK, and LC-3 expressions (arrow) in liver mitochondria extracted from rats without (**A, E, and I**) or with simvastatin (B, F, and J) and sericin (**C, G, and K**) treatments. The expression of these markers was located on mitochondrial cristae, matrix and membrane. Bar graphs compared these expressions among treatment groups (**D, H, and L**). *; *P* ≤ 0.05, **; *P* ≤ 0.01, ***; *P* ≤ 0.001, ****; *P* ≤ 0.0001.

To determine the effect of sericin on CARD-9 and MAPK expression, in association with the liver cell homeostasis, immune-electron microscopic study was performed. In an *in vitro* study, CARD-9 immunolabelling in simvastatin-treated cell was significantly higher than sericin-treated cells in both concentrations (0.125 and 1 mg/ml) (Fig. 2). Contrast to MAPK immunolabelling, sericin-treated cells had significantly higher expression of MAPK than presented in simvastatin-treated cell and non-treated cell (Fig. 3). In agreement with an *in vivo* study, compared to non-treated rat, the expressions of CARD-9 and MAPK were significantly increased in the mitochondria from rat-treated with simvastatin and sericin, respectively (Fig. 4A-H).

### 3.5 Blood cholesterol and histopathological changes in hypercholesterolemic rat

**Fig. 5:**
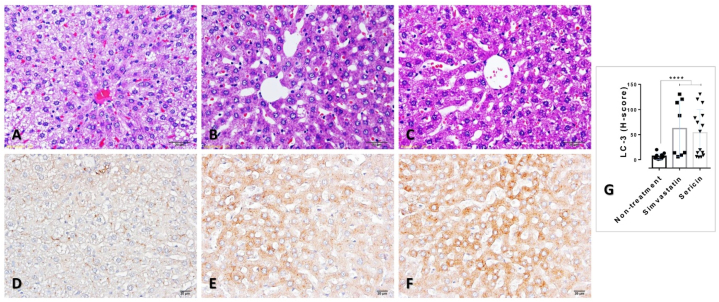
Histopathology and LC-3 immunolabelling in the liver from the rat among treatments: H&E staining indicated the hepatic microvesicular steatosis in liver tissue from rats without treatment (**A**), while the rat with simvastatin (**B**) and sericin (**C**) treatments had improved hepatic conformation. LC-3 immunohistochemical staining demonstrated the down-regulation of LC-3 in non-treated rats (**D**) compared to simvastatin (**E**) and sericin (**F**) treatments. Bar graphs compared these expressions among treatment groups (**G**). *; *P* ≤ 0.05, **; *P* ≤ 0.01, ***; *P* ≤ 0.001, ****; *P* ≤ 0.0001.

After treatment with 7.4 mg/kg/day of simvastatin and 1000 mg/kg/day of sericin extract for 28 days, total blood cholesterol in treated-rats (with simvastatin: 124.89 ± 5.98 mg/dL and with sericin: 145.87 ± 7.58 mg/dL) was significantly lower that non-treated rats (350.85 ± 18.45 mg/dL). Microvesicular steatosis, a small lipid droplet deposition in the hepatic cytosol, was predominately observed in non-treated rats when compared with simvastatin and sericin treatment groups (Fig. 5A–C).

### 3.6 Sericin promotes hepatic autophagy characterized by LC-3 expression

**Fig. 6:**
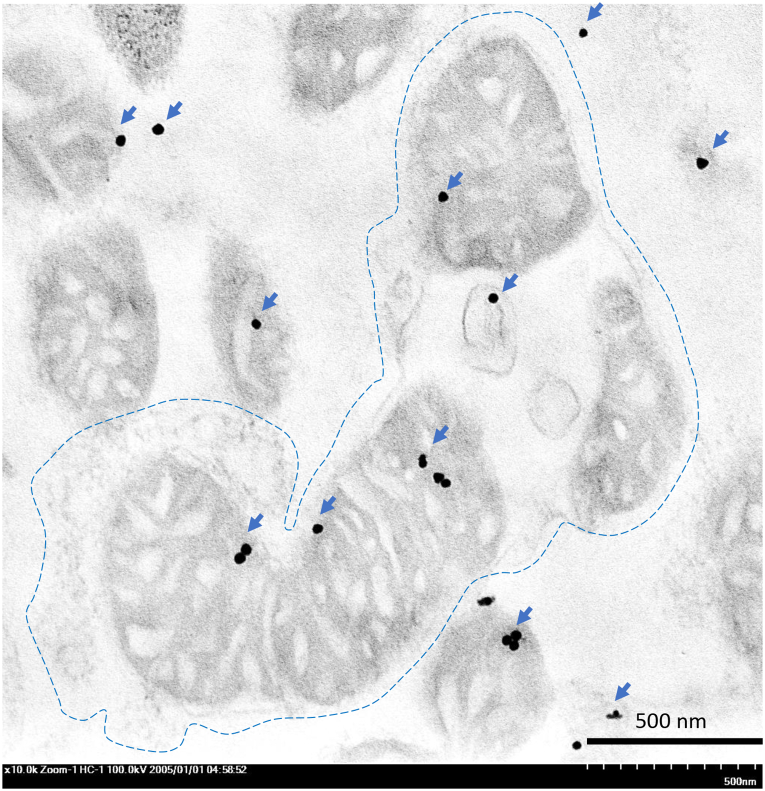
Hepatic mitochondrial autophagy in rat-treated with sericin, labelled with LC-3 gold particles: An electron micrograph exhibited the accumulation of mitochondria in autophagosome (dash-line) with immunolabelling of LC-3 (arrow). Three mitochondria with few cellular debrises were observed in this mitophagic cell. *; *P* ≤ 0.05, **; *P* ≤ 0.01, ***; *P* ≤ 0.001, ****; *P* ≤ 0.0001.

**Fig. 7:**
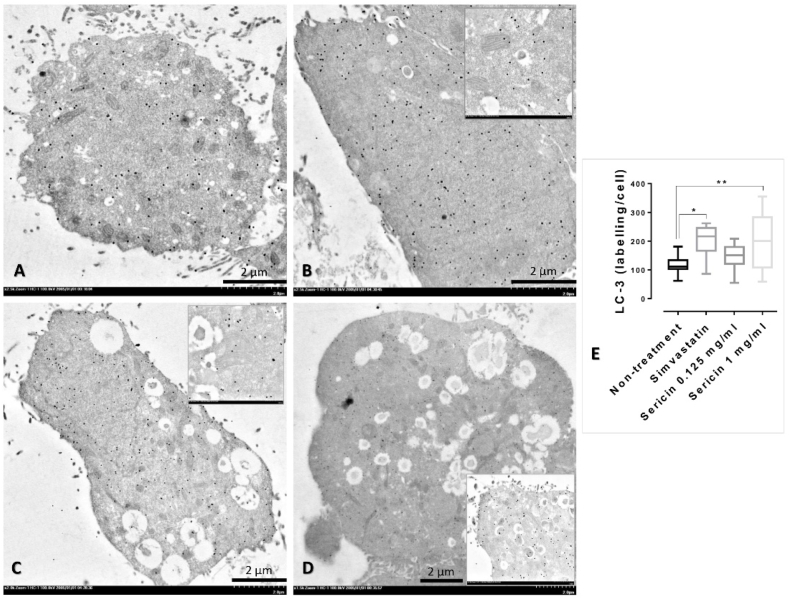
LC-3 immunolabelling in the HepG2 cell among treatments: Electron micrographs shown immunogold labelling of LC-3 expression (gold particle dots) on HepG2 whole cell without (**A**) or with simvastatin (**B**) and sericin (**C-D**) treatments. LC-3 immunolocalization was observed throughout of the HepG2 cell both in the cytoplasmic and nucleolar areas. Inset images represented a higher magnification with the expression on mitochondria and cytosol. Bar graph compared the expression among treatment groups (**E**). *; *P* ≤ 0.05, **; *P* ≤ 0.01, ***; *P* ≤ 0.001, ****; *P* ≤ 0.0001.

Hepatic autophagy in relation to LC-3 expression was verified by immunogold labelling and immunohistochemical techniques. Rat-treated with sericin exhibited significantly higher level of LC-3 expression both in the liver mitochondria and tissue than non-treatment group (Fig. 4, Fig. 5). Electron micrograph also demonstrated a feature of mitochondrial autophagy, which represented by an accumulation of mitochondria in autophagosome with same limited membrane (Fig. 6). In addition, both HepG2-treated with simvastatin and high-dose of sericin had significantly higher level of LC-3 expression in the cell than observed in non-treated cell (Fig. 7).

## 4 Discussion

Apart from our reports [[16], [17], [18], [19], [20], [21], [22],^33^,^34^] that focusing on hypocholesterolemia, anti-inflammatory, anti-melanogenicity, and healing properties of sericin, there are several studies indicated that sericin has its own therapeutic effects on many aspects such as diabetes mellitus [35,36], obesity [37], cardiovascular defect [38], neurological defects [39], and hepatoprotective property [40]. However, the effect of sericin on liver detoxification such as lipid or alcohol toxic metabolites and ammonia remain unclear. The present study demonstrated that sericin promotes the levels of liver detoxification enzyme (CYP1A2 and ALDH-2) and urea cycle enzyme (CPS-1 and OTC) genes. Sericin also enhances hepatic autophagy, which might be an important role to accelerate urea synthesis, as characterized by the increment of LC-3 expression in the liver mitochondria. Along with these findings, a negative correlation between CARD-9 and MAPK expressions was found in the liver mitochondria indicating that sericin promotes ammonia elimination in the liver disclosing to CARD-9/MAPK pathway.

Hyperammonemia from both primary cause by inherited urea cycle disorder and secondary cause such as liver diseases or viral infection leads to several health problems as presented in central nervous system e.g. combativeness, lethargy, and coma, in respiratory system e.g. shortness of breath, and in neuromuscular system e.g. poor coordination, ataxia, tremor, and seizures decerebrate posturing. To eliminate an excess ammonia, urea cycle plays a pivotal role to convert ammonia to urea in periportal hepatocyte belonged to two enzymes in the mitochondria, which are CPS-1 and OTC and three enzymes in the cytosol (argininosuccinate synthetase, argininosuccinate lyase, and arginase). The complete urea cycle also need amino acid transporters and other enzymes involving in nitric oxide, proline, or glutamine synthesizes for its fully function [41]. Regarding to these mechanisms, some medications using to alleviate ammonia toxicity are therefore designed to maintain imperforated urea cycle [1,2]. Although an effective regulation of the urea cycle enzyme levels leads to properly liver ureagenesis, the underline mechanisms still not well-understood. A decrease expression of CPS-1 relates to a reduction of ammonia detoxification in various defects especially in hypercholesterolemia and fatty liver (reviewed in Ref. [41]). In addition, both in rat model [42] and human cases [43] of NAFLD demonstrate the markedly reduction of CPS-1 and OTC activities (gene and protein expressions) leading to the impairment of urea synthesis. Focusing on therapeutic aspect for urea cycle enzymes enhancer, up to date, there are few reportedly substances that have anti-hyperammonemia and hepatoprotective effects in association with improving CPS-1 and OTC gene levels such as bioflavonoid quercetin [44] and *Rana catesbeiana* homologue of C/EBP alpha [45]. Along with these mentioned studies, it could be seen that sericin not only enhances ammonia removal by increase the levels of CPS-1 and OTC genes, the mitochondrial urea cycle enzymes, sericin also promotes liver detoxification property by increases the levels of CYP1A2 and ALDH-2 genes, the cytosolic detoxification enzymes (Fig. 1).

Regarding to caspase and MAPK activities, it has been believed that the relationship (both positive and negative) of these two proteins involving to the occurrence of autophagy, a crucial role for elimination degraded cytoplasmic components, is a high potential role for describing pathogenesis in several diseases. The examples for such studies are mentioned below. Fenofibrate, a peroxisome proliferator-activated receptor-alpha, reduces cisplatin nephrotoxicity majority by the suppression casepase-3, -8, and -9 and MAPK altogether with low involvement of renal autophagy [11]. Caspase-10 deficiency causes low activity of CPS-1 gene in urea cycle leading to promote cellular proliferation via MAPK activity especially in bladder cell cancer [12]. Down regulation of caspase-9 promotes proliferative, invasive, and migrative properties during high signaling of MAPK leading to poor prognosis in non-small cell lung cancer [13]. All-trans retinoic acid alleviates transmissible gastroenteritis virus-induced intestinal apoptosis via caspase-3 and -9 by inhibition MAPK signaling pathway [14]. Lastly, bifendate derivative activates hepatic cell apoptosis via mitochondrial pathway by the induction of MAPK signaling [15]. How autophagy promotes ureagenesis in the liver? The answer is still unclear. Interestingly, it has been reported that hepatic autophagy regulates and monitors liver metabolism, which induced by high amount of blood ammonia [6,46]. Liver autophagy is potentiated by hyperammonemia in association with mammalian target of rapamycin complex 1 inhibition. However, there is no report about an effect of sericin on the link between CARD-9/MAPK signaling pathway and hepatic autophagy regarding to ammonia detoxification. Our explore using immune-electron microscopic study showed a negative relationship of caspase-9 to MAPK expressions in connection with the enhancement of LC-3 expression where significantly high labelling in sericin-treated cell, liver tissue, and liver rat mitochondria (Fig. 2, Fig. 3, Fig. 4, Fig. 5, Fig. 6, Fig. 7). Based on these evidences, therefore it is a high possibility that ureagenesis might be promoted by sericin. However, our experiments have some limitations. In animal study, to better understand the effects of sericin and simvastatin, a healthy control group receiving a normal diet should be explored for clearer comparison. In *in vitro* study, digested sericin, single amino acids, or peptides might be additionally used to apply for HepG2 cell culture to perceive better therapeutic properties of sericin mimicking to its oral route digested form.

In summary, this study revealed the ureagenesis effect of sericin on both *in vitro* and *in vivo* models. Clarification of the mechanism demonstrated by the acceleration capacity of sericin on the level of liver detoxification enzyme and urea cycle enzyme genes in association with hepatic autophagy. Moreover, CARD-9/MAPK pathway contributed to these underling mechanisms. Therefore, this work highlights the possibility of sericin against hyperammonemia and maintains hepatoprotective effect.

## Ethics approval and consent to participate

Animal studies were approved by the Faculty of Medicine, 10.13039/501100002873Chulalongkorn University Animal Care and Use Committee, Bangkok, Thailand (Approval No. 16/2558).

## Consent for publication

Not applicable.

## Availability of data and materials

The datasets used and/or analysed during the current study are available from the corresponding author upon reasonable request.

## Funding

This study was supported by 10.13039/501100017170Thailand Science Research and Innovation Fund, 10.13039/501100002873Chulalongkorn University (CU_FRB65_hea (50)_059_33_03): P.A.; The National Research Council of Thailand: P.A.; 10.13039/501100004156Mahidol University, Basic Research Fund: fiscal year 2024: S.A.

## CRediT authorship contribution statement

**Sumate Ampawong:** Conceptualization, Data curation, Formal analysis, Funding acquisition, Investigation, Methodology, Resources, Validation, Writing – original draft, Writing – review & editing. **Napatara Tirawanchai:** Conceptualization, Formal analysis, Investigation, Methodology, Resources, Supervision, Validation, Visualization, Writing – review & editing. **Tapanee Kanjanapruthipong:** Formal analysis, Investigation, Methodology, Visualization, Writing – review & editing. **Kamonpan Fongsodsri:** Conceptualization, Data curation, Formal analysis, Investigation, Methodology, Validation, Visualization, Writing – original draft, Writing – review & editing. **Khwanchanok Tuentam:** Formal analysis, Investigation, Methodology, Visualization, Writing – review & editing. **Duangnate Isarangkul:** Data curation, Investigation, Methodology, Resources, Supervision, Validation, Visualization, Writing – review & editing. **Pornanong Aramwit:** Conceptualization, Data curation, Formal analysis, Funding acquisition, Investigation, Methodology, Project administration, Resources, Supervision, Validation, Visualization, Writing – review & editing.

## Declaration of competing interest

The authors declare that they have no known competing financial interests or personal relationships that could have appeared to influence the work reported in this paper.
